# P-151. Epidemiology of Vibriosis Cases in Los Angeles County, 2014-2023

**DOI:** 10.1093/ofid/ofaf695.377

**Published:** 2026-01-11

**Authors:** Joy Suh, Marifi Pulido, Dawn Terashita, Sharon Balter, Jemma Alarcón

**Affiliations:** Keck School of Medicine of USC, San Gabriel, CA; Acute Communicable Disease Control Program, Los Angeles, California; Los Angeles County Department of Public Health, Los Angeles, CA; LA County DPH, Los Angeles, California; Los Angeles County Department of Public Health, Los Angeles, CA

## Abstract

**Background:**

Vibriosis caused by non-toxigenic *Vibrio* bacteria is a reportable foodborne illness responsible for 80,000 infections in the United States each year. It is contracted from marine or brackish waters and eating undercooked or raw seafood. In this study, we examined the demographic characteristics and incidence trends of vibriosis in Los Angeles County (LAC) from 2014 to 2023.

**Figure:**
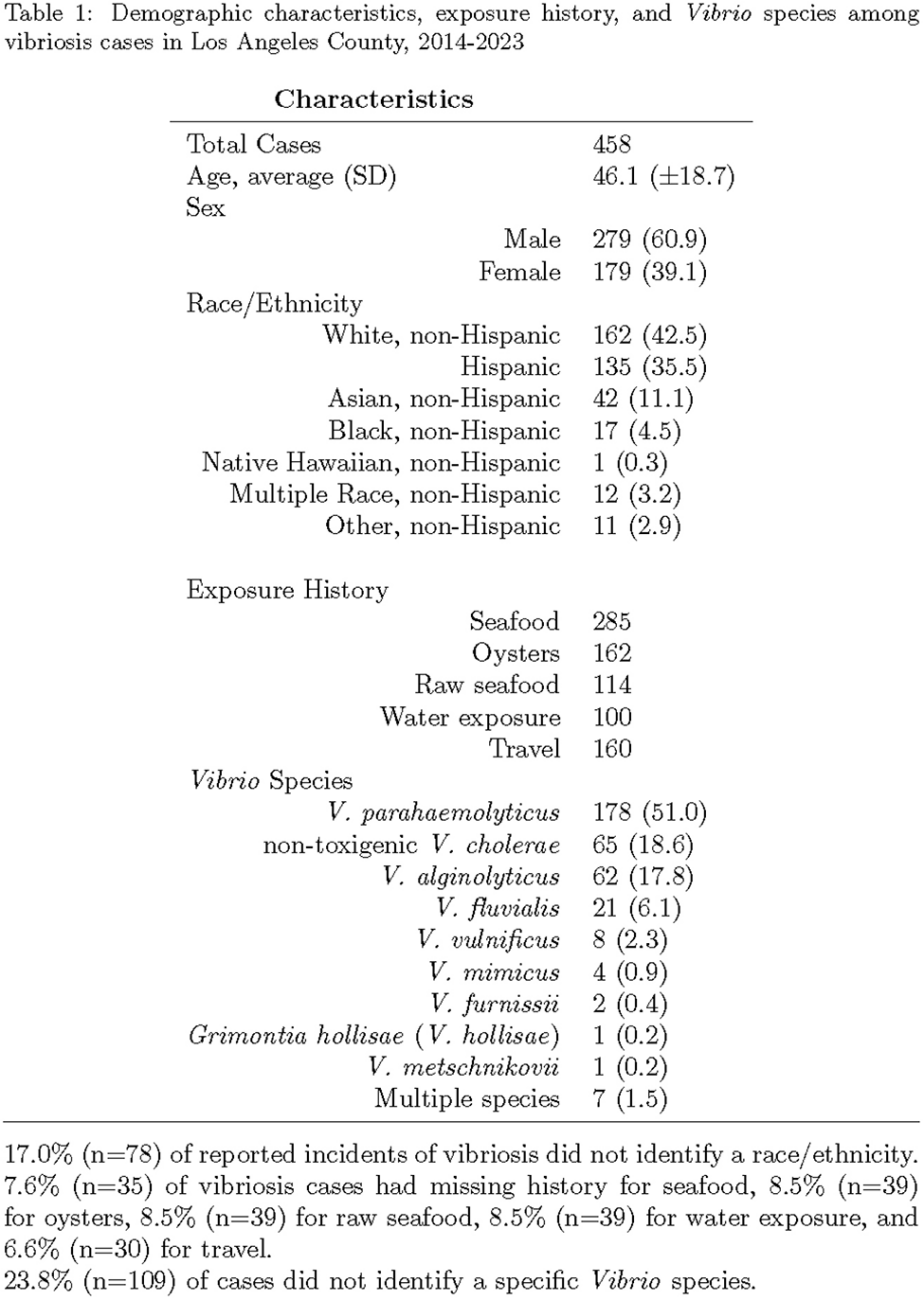

The table summarizes key characteristics of probable and confirmed vibriosis cases including age, sex, race/ethnicity, exposure sources, and the distribution of Vibrio species identified in LAC from 2014 to 2023.

**Figure 1:**
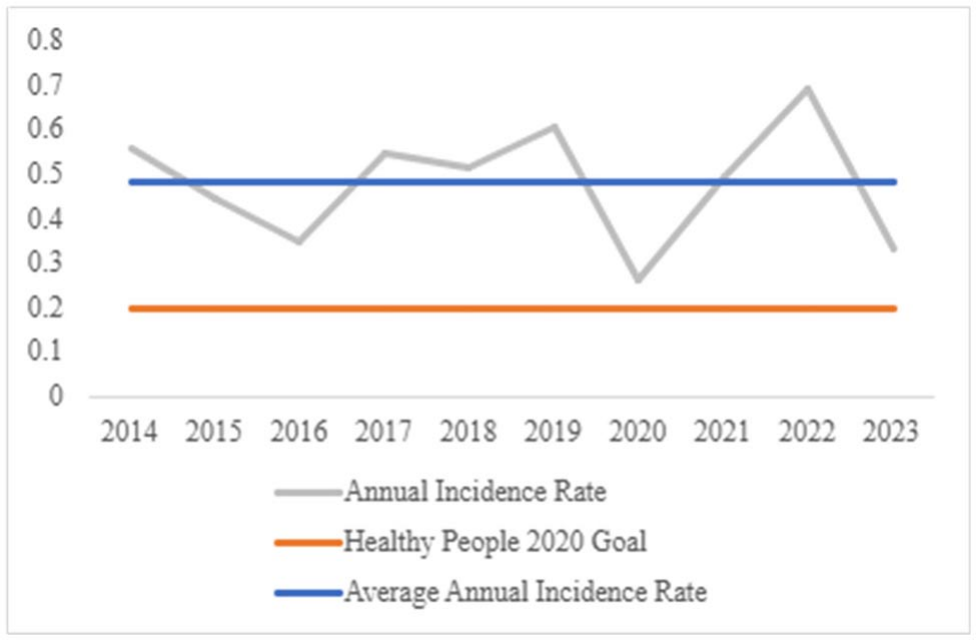
Vibriosis annual incidence rate per 100,000 in Los Angeles County, 2014-2023

The graph depicts annual incidence rates for vibriosis over a ten-year period from 2014 to 2023 in LAC. The average annual incidence rate and Healthy People 2020 goal rate for Vibrio are shown.

**Methods:**

We analyzed confirmed and probable vibriosis cases reported to LAC from January 1, 2014, to December 31, 2023. Using SAS 9.4, we conducted descriptive epidemiological analyses of annual incidence rates, case characteristics (age, sex, race/ethnicity), exposure history, and Vibrio species. Rates were compared to the Healthy People 2020 (HP2020) goal of 0.2 per 100,000.

**Results:**

A total of 458 vibriosis cases (348 confirmed, 110 probable) were reported. The average annual incidence rate of vibriosis was 0.48 per 100,000 with the highest rate in 2022 (0.70 per 100,000). Cases were most common among individuals aged 35-44 years (Table 1). About half of all cases were reported in the three-month period of July-September, with cases peaking in August (96 cases, 21%). The most common species were *V. parahaemolyticus* (183 cases, 40%), non-toxigenic *V. cholerae* (66 cases, 14.4%), and *V. alginolyticus* (63 cases, 13.8%). *V. parahaemolyticus* had the highest average annual rate of 0.19 per 100,000. There were eight cases of *V. vulnificus* of which six (75%) were hospitalized. The case-fatality rate for *V. vulnificus* was 50% (4 deaths). Seafood was the most reported exposure with 56.8% (162 cases) having eaten oysters and 40% (114 cases) raw seafood. The most common international destination was Mexico (54 cases, 31%) and the majority of domestic travel was within CA, outside of LAC (40 cases, 48.8%). Of those with multiple exposures, the most common combination was food or drink and travel (82 cases, 53.3%).

**Conclusion:**

The annual incidence rates for vibriosis in LAC from 2014 to 2023 remained stable and did not meet the HP2020 goal. People who are non-Hispanic white, male, or 35-44 years old appear to be disproportionately affected. With LAC’s average rate being 2.4 times higher than the HP2020 goal, there is a need for continued prevention efforts targeting the consumption of raw or undercooked seafood both domestically and abroad.

**Disclosures:**

All Authors: No reported disclosures

